# Sundown Journalism

**Published:** 1904-10

**Authors:** 


### SUNDOWN JOURNALISM.*

Institutions organized to give medical lectures and instruction only in the evening are called "sundown colleges." The supposition is that a number of young men who are busily occupied during the day with other work are unable to take up medical studies until after sundown, or in the evening. It is claimed that limited means and urgent duties prevent them from studying medicine at any other time; hence this teaching in secondary, and is a form of by-product instruction, after the stress and strains of the day.

I have used this term to describe some of the peculiarities and eccentricities which appear in journals and journalistic work—the simplest explanation of which is sundown work, or work done when the brain and body are debilitated and below par from the strains and labor of the day. Scientific work in the medical journals or elsewhere is rarely demanded under stress and strain, or should be consigned to sundown periods, when the brain is debilitated from other duties and is less clear and vigorous. Critical readers of medical journals are frequently surprised at the wide variation in the quality, style and tone of the work presented. The editorials differ widely and the point of view often changes, and the thought is sometimes harsh, discordant or vague and confused. Credulity and skepticism alternate so rapidly as to confuse the reader. While the editor is known to be a man of excellent judgment and careful in his conclusions, the editorial work fails to confirm this estimate of his ability. The inference is that this is the work of other authors, and my plea last year before this association to have all editorial matter signed, was to enable the reader to clear up this mystery.

*Read by T. D. Crothers, M.D., Hartford, Conn., Editor *Journal of Inebriety*, before the Medical Association at Atlantic City, June 6, 1904.

While journals, like individuals, have frailties, and editors have distinct personalities, the reader is distressed when these peculiarities change rapidly, and become confused and hysterical, and move on unusual zigzag lines. The real pleasure to the reader of a journal is to know and respect the author's consistency, good judgment and uniform impressions of the changing conceptions of science. When he fails in this, evidently some clouds have come into the horizon and broken up the usual order of events—one explanation of which would be sundown journalism—work done under unfavorable conditions, forced work, in which tobacco, coffee, tea, whisky, morphine are appealed to for help. Editorial comments on matters of which we know the author's familiarity which fall so far below the usual level of good sense and clearness of expression, must be attributed to this source.

During the past year, many of the contributed articles on consumption have brought out distinct earmarks and footprints of sundown thinking and working. Evidently some of these papers were written by persons suffering from this disease, with its peculiar delusions, and mental twists, largely influenced by the time of writing and the form of drugs used. Often papers on appendicitis have a marked sundown movement with a confused exhaustive tone. Such writers are working at night suffering from fatigue, strain and exhaustion, following the operations and other work which they have performed.

Most of the great weeklies and some of the monthly journals contain many marked examples of sundown contributions, noted in the jarry, exclamatory style from the effects of alchohol, or the softer notes and the assertive confidence in which conclusions are stated under the influence of morphine. The cocainist influence in these contributions is more pronounced than that of any other drug, particularly in the endless repetitions and exclamations involved, and movement in a dreamy, hazy mass of words.

The query is often made why all this mass of journalistic work coming from the press every week and month should be so ephemeral and valueless. The evident explanation of it is sundown writing, stimulated by drugs, and efforts to work the body and brain at a time when rest is required.

The same error appears in books. Often a clear, vigorous medical teacher, whose work in the classroom is stimulating, will write a book that utterly fails to sustain his reputation. The impression in the classroom as a teacher is broken up by the halting, obscure, non-stimulating, expressionless work in the volume. Reviewers are disappointed in the text-books of eminent practitioners and teachers, that fail to present the best thought and conclusions, and practically are nothing but involved literary patchwork, lacking in form, shape and vigor. The critic is conscious that the author has given only a weak production of what he could have done; hence his praise is formal and mechanical. One of the popular text-books on the market is notoriously a midnight work, stimulated by opium and cocaine; another text-book with a large sale has drug writing and drug work on every page, and the so-called brilliant passages are followed by vague statements so marked that the exact drug used can almost be pointed out.

Frequently some eminent physician in active practice will deliver an address as president or orator for some occasion, and the expectations of his friends will be greatly disappointed. The vigorous, clear-headed man when seen in print appears as a vague, sophomoric thinker, confused in range of thought, jarring in style and seldom rising to the time and occasion. Often the effort to be strictly scientific appears most prominent by following other authors and restating their conclusions from different points of view, dovetailed with his own opinions, and all covered with an air of mystery and technical words.

Lectures, essays and pamphlets which conclude with an enormous bibliography, convey the impression and expectancy of an exhaustive effort; yet, when critically examined, the sundown flavor and general feebleness of work is apparent. German literature is very often marred by these posing effects of bibliography following commonplace writing on well-known subjects, mixed up with excessive technicalities and sentences so involved that the author's meaning is never clear.

Editors are always troubled with contributions from influential, active medical men whose writings are mere by-products and feeble sundown efforts. Their standing in the community and reputation make it difficult to refuse such work, and yet the editor knows that it is mere stuff and fustian, and often without the merit of style and culture. The only objection that can be offered is that the columns are crowded and his stock of supplies exceeds the demand for a long time to come, and this is literally true of this class of work. I have come in contact with many medical men, noted as voluminous writers, who are, or have been, disabled from the use of spirits and drugs. They usually suffer from insomnia and take to writing midnight articles that reflect accurately their exact mental condition. Some of these writers after varied experience as journal contributors become book authors. It is needless to add that the paranœic brain, influenced by beer, alcohol and drugs, appear more or less prominently in all their work. Foreign medical literature both in journals and books often exhibit the same marked traits, the same banquet twist, the same wine and beer coloring, and the same midnight work often unmistakable. While the range of thought is less versatile and vigorous, it always excels in stupid conservatism and technical minutia.

To all careful observers, the examples of this kind of work apparent in general literature will be surprising. Magazines and articles and books present many illustrations of midnight work done under the influence of alcohol, morphine and cocaine. A book having a large sale and admired by many persons was written by one using cocaine. The plots, the mystical style and the range of thought and other indications are conclusive. It is surprising to note how exactly the writer, both in scientific and general literature, unconsciously and exactly describes his mental health and mental condition in his writings.

As editors and writers, we may not always be able to make wise discriminations of the contributions offered or determine the real value of scientific writing, but personally we can avoid sundown work and midnight thinking, and thus cultivate sharper eyes and clearer brains in detecting the movement and direction of scientific progress. An article recently published by a very clever critic and medical man claims that much of the literature of today can be aptly characterized as the direct products of coffee, beer, wine, spirits and compounds of opium and cocain. There can be no doubt this is true to some extent. We all realize that no one can think or write clearly on any subject with a weary brain, or one forced into service by drugs. It is difficult to understand how a medical man can expect, after the labors and duties of the day, to retire to his office at night and do any good scientific literary work. Medicine and literature in any form or direction to be successful requires the clearest thought and the best energies under the most favorable conditions.

Journals, editors, and authors can never bring out forced sundown products and become successful workers. Journals die of neglect and dementia, simply because they are the products of fatigue and forced, unnatural efforts. Editors become marasmic and disappear, because their best energies and efforts have been diverted in other directions, and their literary work is only by-products. Authors pose for a little time and then are forgotten, because they had no message and no thought to communicate. What they said was thought after sundown in an atmosphere of tobacco, coffee, spirits and drugs. In all this there was mental and physical starvation, acute mental poverty, with consequent confusion of thought and purpose. I repeat what I have said before—that these obvious conditions explain much of the failures in journalism and literary and scientific efforts by medical men. The surgeon, the neurologist and the active practitioner in every department of medicine can never write lectures, learned essays, or clear editorials after sundown. Such work must be done in the morning under the very best conditions and favorable circumstances for clear thinking and exact writing. Dictating to a stenographer after dark when the duties of the day are ended carries with it a distinct impression which critical readers do not fail to discover. Sundown books, sundown authors, and sundown journals ought to disappear and will do so in the near future. When journals appear to meet a real demand, and editors understand the conscious and unconscious claims of scientific medicine, and authors feel that they have a real message and group of facts to communicate, then the mediocrity of medical and particular journalistic literature will pass away.—*Virginia Med. Semi–Monthly.*

